# A simple and practical approach for protein serial crystallography using grease matrix and large-area support film

**DOI:** 10.1107/S1600577526005643

**Published:** 2026-07-15

**Authors:** Michihiro Sugahara, Saori Maki-Yonekura, Ichiro Inoue, Kiyofumi Takaba, Shun Narai, Hisashi Naitow, Jungmin Kang, Kensuke Tono, Keiji Numata, Tetsuya Ishikawa, Makina Yabashi, Koji Yonekura

**Affiliations:** a RIKEN SPring-8 Center, 1-1-1 Kouto, Sayo-cho, Sayo-gun, Hyogo679-5148, Japan; b Sumitomo Pharma Co. Ltd, Konohana-ku, Osaka554-0022, Japan; c Japan Synchrotron Radiation Research Institute 1-1-1 Kouto, Sayo-cho Sayo-gun Hyogo679-5198 Japan; d Biomacromolecules Research Team, RIKEN Center for Sustainable Resource Science, 2-1 Hirosawa, Wako, Saitama351-0198, Japan; e Department of Material Chemistry, Graduate School of Engineering Kyoto University Kyoto615-8510 Japan; f Institute of Multidisciplinary Research for Advanced Materials Tohoku University Sendai Aoba-ku Japan; POSTECH, Republic of Korea

**Keywords:** serial crystallography, fixed target, large-area sample support, grease matrix, X-ray free-electron laser (XFEL)

## Abstract

Serial femtosecond crystallography (SFX) enables structural determination of biological macromolecules at room temperature using ultrashort X-ray pulses from an X-ray free-electron laser, thereby minimizing radiation damage and capturing dynamic structural changes. Efficient sample delivery is essential for collecting many diffraction patterns. The grease matrix injection method reduces sample consumption but may encounter nozzle clogging and unstable flow when the crystal size is large, the crystal density is high, or the viscosity of the crystallization buffer-grease mixture increases. Fixed-target approaches, in which crystals are dispersed on a support film, offer a stable alternative with lower sample consumption. Here, we present an optimized fixed-target system integrating a grease matrix and a large-area support film, enhancing sample stability, data collection efficiency, and structural resolution while enabling potential hydrogen atom detection in SFX experiments.

## Abstract 2

A simple fixed-target protein serial crystallography method combining a grease matrix with a large-area support film enables stable sample handling, low sample consumption, and high-resolution room-temperature structure determination.

## 1. Introduction

Serial femtosecond crystallography (SFX) using X-ray free-electron laser (XFEL) pulses (Emma *et al.*, 2010 ▸; Chapman *et al.*, 2011 ▸; Ishikawa *et al.*, 2012 ▸; Barty *et al.*, 2012 ▸; Schlichting & Miao, 2012 ▸) offers a means to overcome typical radiation damage to samples such as proteins (Liu *et al.*, 2013 ▸; Redecke *et al.*, 2013 ▸; Cohen *et al.*, 2014 ▸; Hirata *et al.*, 2014 ▸; Hunter *et al.*, 2014 ▸; Weierstall *et al.*, 2014 ▸; Sugahara *et al.*, 2015 ▸; Kang *et al.*, 2015 ▸; Colletier *et al.*, 2016 ▸) and chemical compounds (Schriber *et al.*, 2022 ▸; Takaba *et al.*, 2023 ▸; Støckler *et al.*, 2023 ▸; Takaba *et al.*, 2024 ▸; Kotei *et al.*, 2024 ▸; Kang *et al.*, 2024 ▸). This is achieved through the ‘diffraction-before-destruction’ approach (Neutze *et al.*, 2000 ▸), in which numerous crystals are sequentially irradiated with short XFEL pulses at a high repetition rate. Thus, SFX has opened new opportunities for femtosecond–microsecond-scale time-resolved studies of structural changes and chemical dynamics (Barends *et al.*, 2015 ▸; Nango *et al.*, 2016 ▸; Pande *et al.*, 2016 ▸; Suga *et al.*, 2017 ▸; Tosha *et al.*, 2017 ▸; Coquelle *et al.*, 2018 ▸; Nogly *et al.*, 2018 ▸; Oda *et al.*, 2021 ▸; Maestre-Reyna *et al.*, 2022 ▸; Hosaka *et al.*, 2022 ▸; Murakawa *et al.*, 2022 ▸; Wolff *et al.*, 2023 ▸; Maestre-Reyna *et al.*, 2023 ▸; Safari *et al.*, 2023 ▸; Li *et al.*, 2024 ▸). Various sample delivery methods for SFX have been reviewed (Martiel *et al.*, 2019 ▸; Echelmeier *et al.*, 2019 ▸; Grünbein & Kovacs, 2019 ▸; Park & Nam, 2023 ▸), highlighting the importance of low sample consumption, a stable sample scan rate synchronized with XFEL pulses, and prevention of crystal degradation, particularly maintenance of the native state of protein samples during the experiments. There are two major types of sample delivery (Martiel *et al.*, 2019 ▸; Echelmeier *et al.*, 2019 ▸; Grünbein & Kovacs, 2019 ▸): (i) injection methods (DePonte *et al.*, 2008 ▸; Weierstall *et al.*, 2014 ▸; Shimazu *et al.*, 2019 ▸) and (ii) fixed-target methods (Zarrine-Afsar *et al.*, 2012 ▸; Hirata *et al.*, 2014 ▸; Hunter *et al.*, 2014 ▸; Cohen *et al.*, 2014 ▸; Mueller *et al.*, 2015 ▸; Lee, Park *et al.*, 2020 ▸). Hybrid delivery methods such as tape-drive droplet injector (Fuller *et al.*, 2017 ▸), crystal extractor (Mathews *et al.*, 2017 ▸) and micro-tubing reeling system (Kim *et al.*, 2024 ▸) have also been introduced.

For protein SFX using the injection method, a liquid jet (DePonte *et al.*, 2008 ▸) containing small crystals is injected at a relatively high speed of ∼10 m s^−1^, consuming at least 10–100 mg of protein. To reduce the sample consumption, micro-extrusion techniques utilizing viscous carrier media, such as a lipidic cubic phase (LCP) (Weierstall *et al.*, 2014 ▸; Fromme *et al.*, 2015 ▸), grease (Sugahara *et al.*, 2015 ▸; Sugahara *et al.*, 2016 ▸; Sugahara *et al.*, 2017 ▸; Sugahara *et al.*, 2020 ▸), Vaseline (petroleum jelly) (Botha *et al.*, 2015 ▸) and hydro­gels (Conrad *et al.*, 2015 ▸), *etc*. (Kovácsová *et al.*, 2017 ▸; Martin-Garcia *et al.*, 2017 ▸; Park *et al.*, 2019 ▸; Nam, 2022 ▸), have been adopted. In addition to these continuous injection approaches, alternative methods have also been reported, such as microfluidic electrokinetic sample holder (MESH) injection, which enables electrokinetic transport of microcrystals, and droplet-based injection systems, which deliver crystals in discrete droplets with reduced sample consumption (Manna *et al.*, 2025 ▸). The micro-extrusion method with these viscous media enables a stable stream at low flow rates of 0.02–0.6 µl min^−1^, reducing sample consumption to less than ∼1 mg. Thus, viscous carrier media have proven to be highly adaptable for protein SFX, accommodating a wide variety of soluble and membrane proteins (Sugahara *et al.*, 2015 ▸; Nakane *et al.*, 2015 ▸; Yamashita *et al.*, 2015 ▸; Sugahara *et al.*, 2016 ▸; Fukuda *et al.*, 2016 ▸; Colletier *et al.*, 2016 ▸; Nakane, Hanashima *et al.*, 2016 ▸; Edlund *et al.*, 2016 ▸; Sugahara *et al.*, 2017 ▸; Suga *et al.*, 2017 ▸; Yamashita *et al.*, 2017 ▸; Naitow *et al.*, 2017 ▸; Hutchison *et al.*, 2017 ▸; Woitowich *et al.*, 2018 ▸; Maestre-Reyna *et al.*, 2022 ▸; Maestre-Reyna *et al.*, 2023 ▸; Li *et al.*, 2024 ▸). However, sample injection through a thin injector nozzle often encounters clogging issues with the sample crystals. To avoid clogging during injection, crystal-size filtering is required (Martiel *et al.*, 2019 ▸). Unfortunately, this filtering step approach results in significant sample loss, increasing sample consumption. Moreover, crystal clogging and/or variations in crystal size lead to unstable flow rates (scan speeds) of the sample stream, which often interrupt diffraction measurement and cause significant difficulties. This is one of the challenges in time-resolved SFX experiments (Kovácsová *et al.*, 2017 ▸). In addition, although injection methods involve experimental complexities such as nozzle clogging, a more fundamental issue is the substantial sample consumption resulting from the waste of crystals between XFEL pulses.

The fixed-target method is more straightforward to set up and operate for SFX, as the crystals are simply placed on a substrate such as traditional goniometer-based pins (Hirata *et al.*, 2014 ▸; Cohen *et al.*, 2014 ▸), silicon chips (Zarrine-Afsar *et al.*, 2012 ▸; Hunter *et al.*, 2014 ▸; Mueller *et al.*, 2015 ▸; Murray *et al.*, 2015 ▸; Oghbaey *et al.*, 2016 ▸; Tolstikova *et al.*, 2019 ▸) or thin films of polyimide (Baxter *et al.*, 2016 ▸; Doak *et al.*, 2018 ▸; Monteiro *et al.*, 2019 ▸; Lee, Lee *et al.*, 2020 ▸; Nam *et al.*, 2021 ▸), and scanned by XFEL pulses. Compared with injection methods, the fixed-target approach avoids measurement interruptions caused by nozzle clogging or stream instability, allowing the sample stage to be scanned continuously at a constant motor speed while also reducing sample consumption. Additionally, the fixed-target method has become an important technique not only for XFEL experiments, where ultrafast structural dynamics are captured, but also for synchrotron-based studies, including time-resolved experiments on the millisecond to second timescale (Schulz *et al.*, 2018 ▸; Weinert *et al.*, 2019 ▸; Aumonier *et al.*, 2020 ▸). The fixed-target methods are categorized into two groups (Martiel *et al.*, 2019 ▸): (i) multi-shot goniometer-based approaches (Hirata *et al.*, 2014 ▸; Cohen *et al.*, 2014 ▸; Suga *et al.*, 2019 ▸), which expose multiple locations of large single crystals (*e.g.* over ∼50 µm) with controlled rotation, and (ii) multiple small-crystal approaches (Zarrine-Afsar *et al.*, 2012 ▸; Hunter *et al.*, 2014 ▸; Baxter *et al.*, 2016 ▸; Doak *et al.*, 2018 ▸; Tolstikova *et al.*, 2019 ▸), which move a sample holder with dispersed small crystals to expose a fresh single crystal to each XFEL pulse. The multiple small-crystal approach is particularly well suited for SFX experiments, as there are fewer limitations in crystal sizes ranging from a few to more than 50 µm in size. For protein SFX using the multiple small-crystal approach, the use of a sheet-on-sheet (SOS) sandwich structure has been reported to prevent damage caused by crystal dehydration (Mueller *et al.*, 2015 ▸; Oghbaey *et al.*, 2016 ▸; Rabe *et al.*, 2020 ▸). This structure utilizes nylon mesh (Lee *et al.*, 2019 ▸; Park *et al.*, 2020 ▸) and hydro­gel media (gelatin and agarose) (Lee, Lee *et al.*, 2020 ▸) to support crystals between the two polyimide films, thereby preventing sinking due to gravity.

Our group has demonstrated a hybrid approach in which a two-dimensional (2D) scan of the substrate coupled with rotation, enabling highly efficient data collection from small organic molecular crystals (Takaba *et al.*, 2023 ▸; Morimoto *et al.*, 2023 ▸; Takaba *et al.*, 2024 ▸; Higashino *et al.*, 2024 ▸). In these studies, we used low-viscosity liquid paraffin to distribute and adhere sample crystals onto a flat-faced polyimide plate with a size of 4 mm × 4 mm. The sample plate was then mounted vertically onto a sample stage equipped with a pin for XFEL exposures. This method is compatible with various crystal types, including small but thick crystals, preferentially oriented plate-like crystals, and crystals ranging in size from a few to over 50 µm, making it particularly well suited for SFX experiments.

Despite these advances described above, sample preparation for protein SFX remains time-consuming, and more efficient, adaptable methods are essential. In this study, we introduce a grease matrix technique into fixed-target protein SFX to further enhance sample delivery. Our method simplifies sample preparation by mixing crystals with a grease matrix medium. Loading the sample involves merely distributing the grease matrix over a large-area polyether ether ketone (PEEK) film. A viscous matrix not only helps maintain crystal distribution by reducing crystal sinking but also appears to prevent crystal dehydration. Crystal orientations within the thick grease layer are random, similar to those in the injection method. Using the grease matrix and a newly developed sample holder for the PEEK film, we successfully determined the structure of proteinase K from *Engyodontium album* at 1.2 Å resolution.

## 2. Results and discussion

### 2.1. Sample preparation, data collection and structure analysis

**Figure 1:**
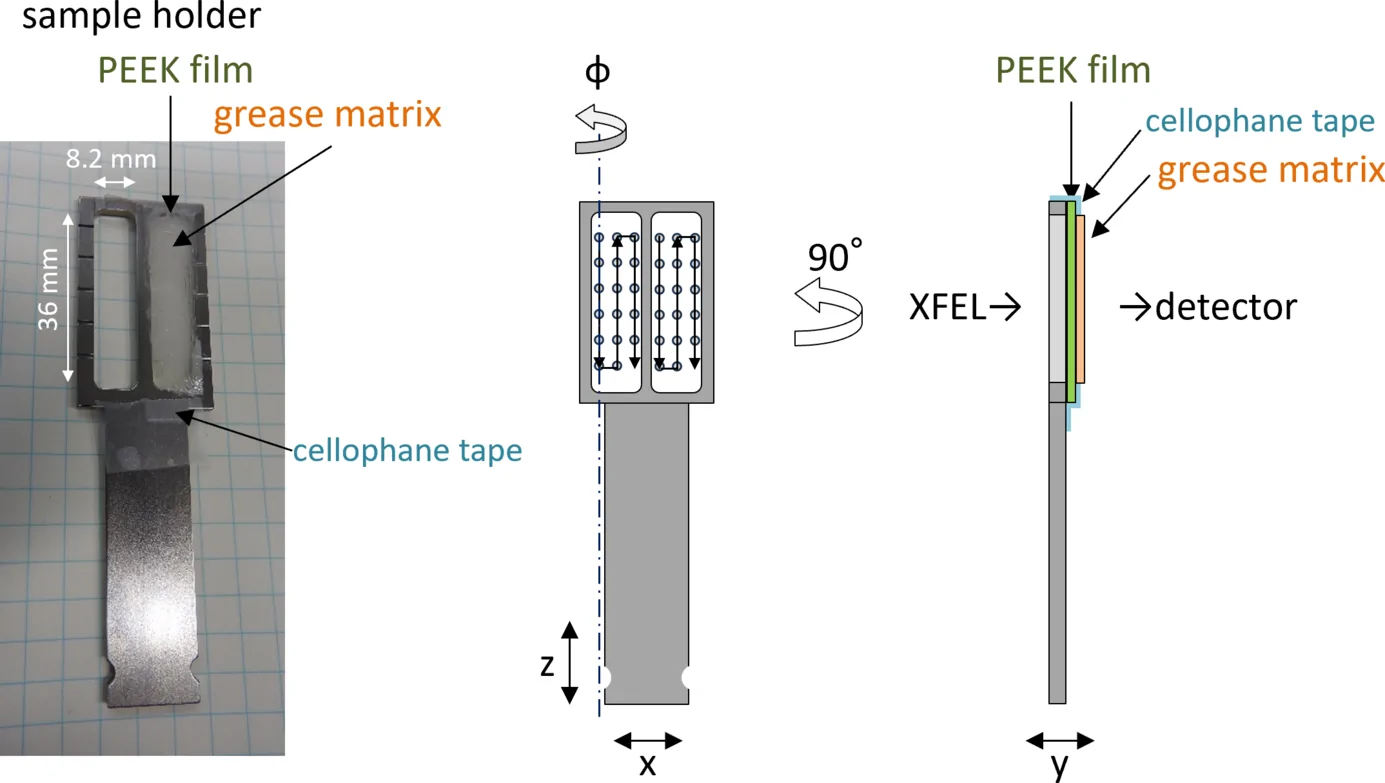
Sample holder. The photograph shows a window with an attached PEEK film coated in a grease matrix. The PEEK film is mounted onto the sample holder using cellophane tape. The illustration depicts the 2D scanning of sample windows with XFEL pulses.

Our previous setup utilized a custom-designed polyimide plate measuring 4 mm × 4 mm and 20 to 40 µm in thickness (Takaba *et al.*, 2023 ▸; Takaba *et al.*, 2024 ▸), which was scanned in 10 µm steps between sequential XFEL exposures in air, without the use of a vacuum path or helium gas. A larger step would be more suitable for protein crystals that are highly sensitive to radiation (Hirata *et al.*, 2014 ▸) and would require a larger area for efficient data collection and time-resolved studies. Increasing the spacing reduces radiation damage and the influence of the pump laser but also increases sample consumption. In this study, as a practical compromise, we used a step size of 25 µm with a total film size of 8.2 mm × 36 mm (Fig. 1 ▸) and a data collection area of approximately 4 mm × 20 mm, which may serve as a useful guideline for future sample preparation and experimental setup. In practice, the primary limitation is the XFEL pulse repetition rate. At very high pulse rates (significantly exceeding 100 Hz), target motion becomes a limiting factor (Moreno-Chicano *et al.*, 2019 ▸). For time-resolved crystallography, recent studies have reported structure determination using this method for protein crystals (Shimada *et al.*, 2017 ▸; Suga *et al.*, 2019 ▸; Bolton *et al.*, 2024 ▸; Gotthard *et al.*, 2024 ▸) and metal organic frameworks (Kang *et al.*, 2024 ▸). Because low-background polyimide film is expensive for large-area applications, PEEK film was used instead, which is substantially more cost-effective, being over 1000 times cheaper than polyimide while maintaining low background scattering suitable for diffraction measurements. Under comparable film thickness and measurement geometry, the background scattering from PEEK is approximately 1.3 times higher than that from polyimide (Hasegawa *et al.*, 2021 ▸). PEEK was selected due to its high thermal resistance (up to ∼250°C) (Mylläri *et al.*, 2015 ▸), chemical stability against acids, bases, and organic solvents, and low dielectric constant (∼3.2 at 1 MHz) (Zhang *et al.*, 2020 ▸), which makes it suitable for stable operation near electronic components. In addition, the film exhibits excellent mechanical strength and produces minimal X-ray background scattering under experimental conditions (Thorne *et al.*, 2003 ▸). A practical advantage of PEEK is that it can be easily cut and processed with scissors, enabling flexible and rapid sample preparation. These combined features make PEEK a highly suitable material for fixed-target crystallography experiments. Our newly developed sample holder features two windows, each of which provides a 2D scan area (Fig. 1 ▸). The frame of the device was fabricated from stainless steel owing to its excellent mechanical strength, corrosion resistance, and chemical stability. Additionally, its ease of machining facilitates prototyping and adjustment.

**Figure 2:**
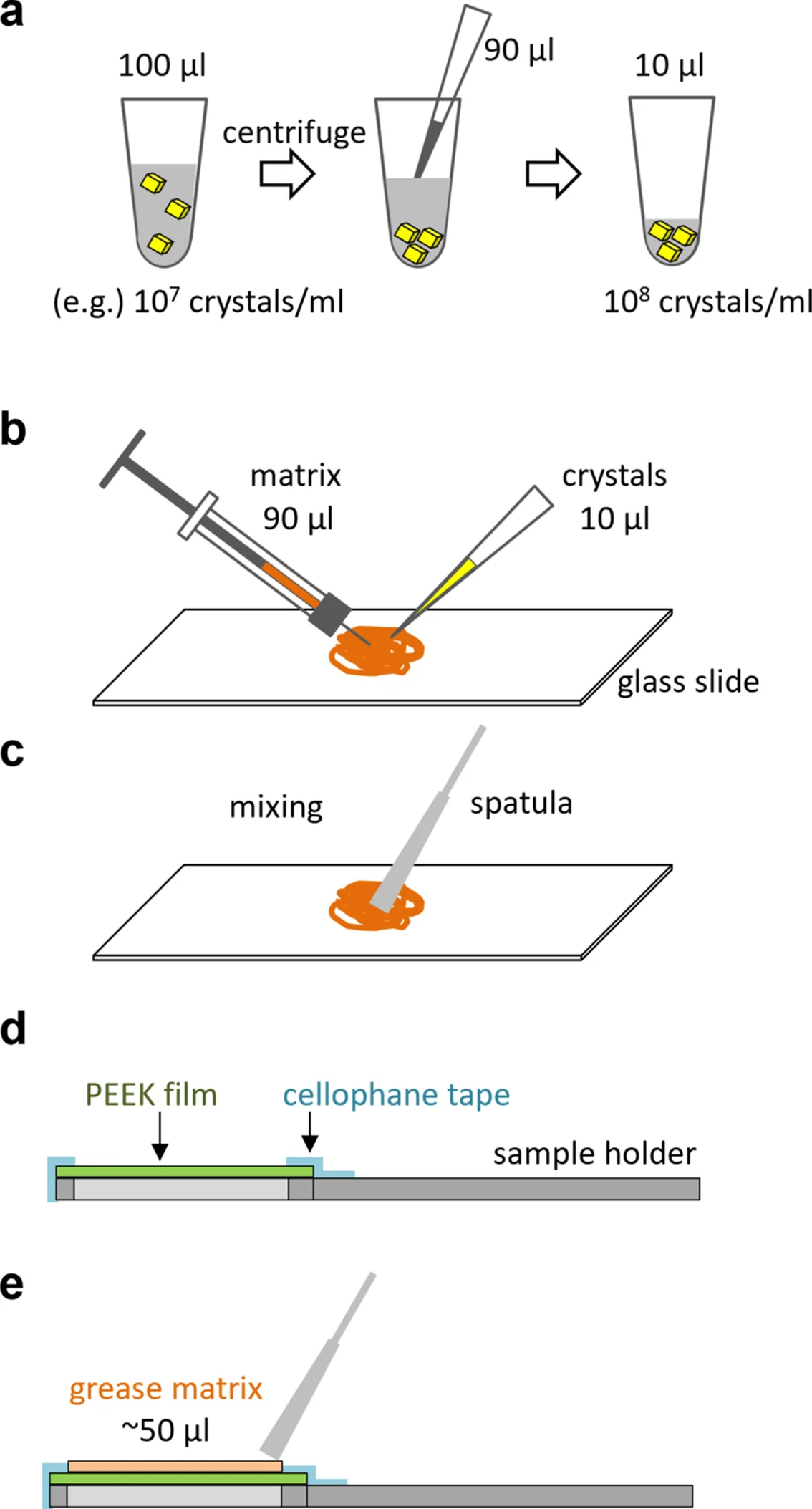
Flowchart of the sample preparation process for the grease matrix technique. (*a*) After centrifuging the crystal sample in the storage solution, the supernatant is removed. (*b*, *c*) The grease and crystal solution are dispensed onto a glass plate and mixed. (*d*, *e*) The grease matrix is spread on the PEEK film, which is mounted onto the sample holder using cellophane tape.

**Figure 3:**
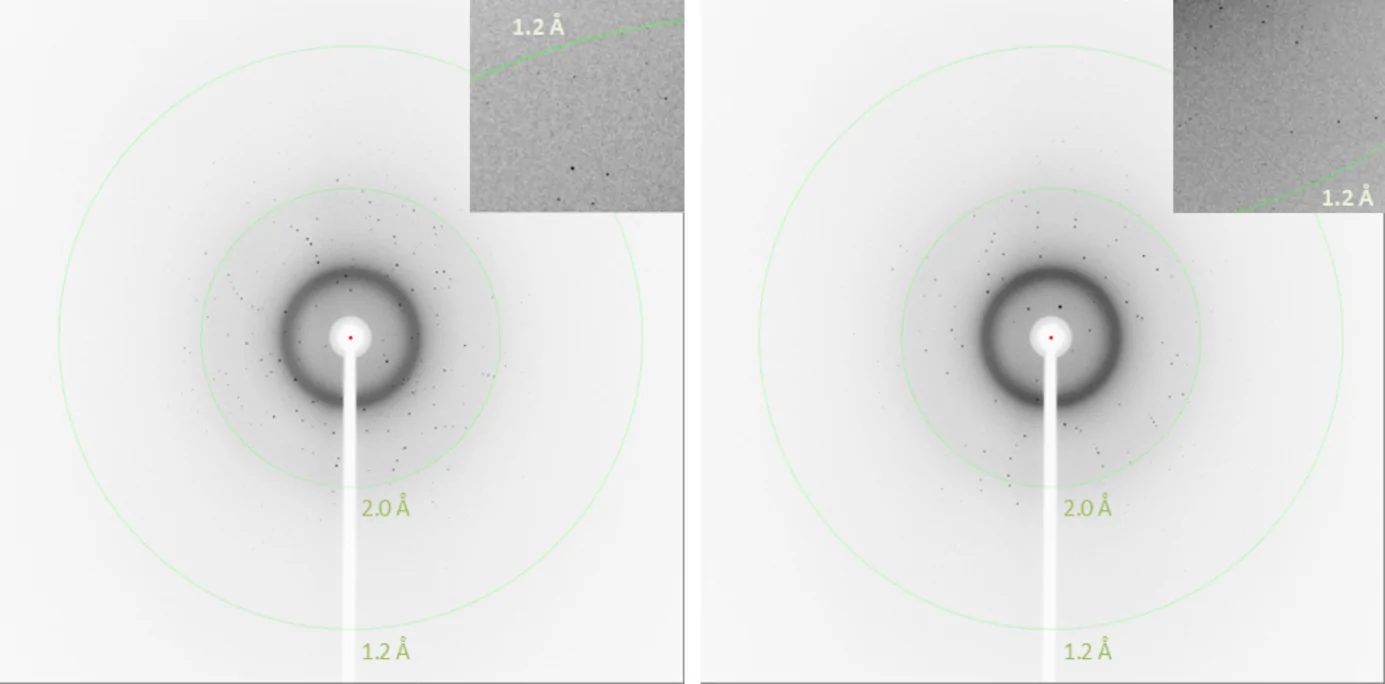
Typical XFEL single diffraction images.

Europium (Eu)-derivatized proteinase K crystals measuring 10 µm × 10 µm in size were then mixed with a grease matrix composed of 15% (*w*/*w*) dextrin palmitate/DATPE grease (DATPE grease) (Sugahara *et al.*, 2020 ▸) and uniformly spread over a PEEK film covering the window of the sample holder (Fig. 2 ▸). Although praseodymium (Pr) had been used previously (Sugahara *et al.*, 2017 ▸; Sugahara *et al.*, 2020 ▸), Eu was used in this study to demonstrate that heavy atoms other than Pr can also provide sufficient anomalous dispersion for SAD phasing. The grease matrix used for sample immobilization was a viscosity-adjustable, hydro­carbon-based grease previously reported in our earlier study (Sugahara *et al.*, 2020 ▸). It was selected for its high compatibility with a wide range of protein crystals and its ability to prevent sedimentation or aggregation over time. Its viscosity allows for smooth spreading onto the support film while maintaining sufficient mechanical stability during data collection. In the fixed-target configuration, the grease was adjusted to a handling-friendly viscosity to prevent crystal displacement due to gravity or X-ray exposure. Additionally, the grease serves as a partial barrier to air exposure, helping to suppress crystal dehydration during the experiment. Notably, it exhibits minimal X-ray background scattering, making it suitable for acquiring high-quality diffraction data. This setting allowed for approximately three times more points to be shot without human supervision compared with the previous setting [(8200 × 36000/25/25)/(4000 × 4000/10/10) ≃ 2.95]. The wavelength of XFEL was selected as 0.827 Å for efficient SAD phasing, to leverage Eu absorption (*f*′ = −0.08 and *f*′′ = 4.76). We collected ∼115000 diffraction images from the PEEK film over one window using a Rayonix MX300-HS detector in about 90 min (Fig. 3 ▸). DATPE grease exhibits background scattering comparable with that of nuclear grease commonly used in SFX (Sugahara *et al.*, 2020 ▸). Compared with hydro­philic media such as cellulose, DATPE grease provides lower background scattering in the low-resolution region, whereas cellulose exhibits lower background scattering in the high-resolution region (above ∼3 Å). Nevertheless, hydro­philic media are prone to dehydration upon exposure to air and are therefore not suitable for the present method. A total sample volume of about 12 µl was used for this experiment, with a crystal number density (after mixing with the matrix) of 1.4 × 10^8^ crystals ml^−1^. We successfully indexed and integrated ∼21000 images for the proteinase K crystals (space group *P*4_3_2_1_2) yielding a 100% complete dataset with a CC_1/2_ of 0.940 at a resolution of 1.2 Å (Table 1 ▸).

In a previous study, the structure of proteinase K was determined using a cellulose hydro­gel matrix in the injection method (Masuda *et al.*, 2017 ▸), achieving a resolution of 1.20 Å with ∼82000 crystals (Table 2 ▸). In this study, we used only ∼21000 crystals to obtain a dataset at the same resolution of 1.20 Å (Table 1 ▸). Data collection statistics indicated no significant discrepancies between the two methods, with a CC_1/2_ of 0.47 and 〈*I*/σ(*I*)〉 values ranging from 1.3 to 1.4 in the outermost shell at 1.22–1.20 Å resolution (Tables 1 ▸ and 2 ▸). However, the experimental conditions for this fixed-target study differed from those of the injection case, which employed the cellulose matrix (Sugahara *et al.*, 2017 ▸) and the MPCCD detector (Kameshima *et al.*, 2014 ▸) at a wavelength of 0.95 Å (Masuda *et al.*, 2017 ▸). Because the *CrystFEL* versions used for data processing differ between studies, and experimental conditions such as crystal size and the presence of heavy atoms are not identical, a strict comparison is not appropriate. The purpose of this study was not to perform a rigorous comparison but to demonstrate that a 1.2 Å structure can be determined using the present method.

The hit rate (27%) and index rate (68%) achieved in this study were comparable with those of the injection method with crystal carrier matrices (Table 2 ▸) (Sugahara *et al.*, 2017 ▸; Sugahara *et al.*, 2020 ▸). Although a ∼50 µl matrix (∼1 mg of protein) was applied to the surface of the PEEK film, we obtained a full dataset by scanning a portion of the film corresponding to a 12 µl aliquot of the matrix (∼0.2 mg protein). In contrast, the continuous sample flow at relatively low flow speed (typically 0.02–0.6 µl min^−1^) for the injection method consumes ∼1 mg or less of the sample (Table 2 ▸) (Shimazu *et al.*, 2019 ▸). Thus, this fixed-target approach can reduce the sample amount to one-third to one-fifth of that in the injection method. The advantage is even more pronounced when considering the crystal-size filtering required in the injection method. The scan speed in both methods appears well suited for XFEL pulses with repetition rates of 30–60 Hz at the SACLA facility.

### 2.2. Crystal structure of proteinase K

**Figure 4:**
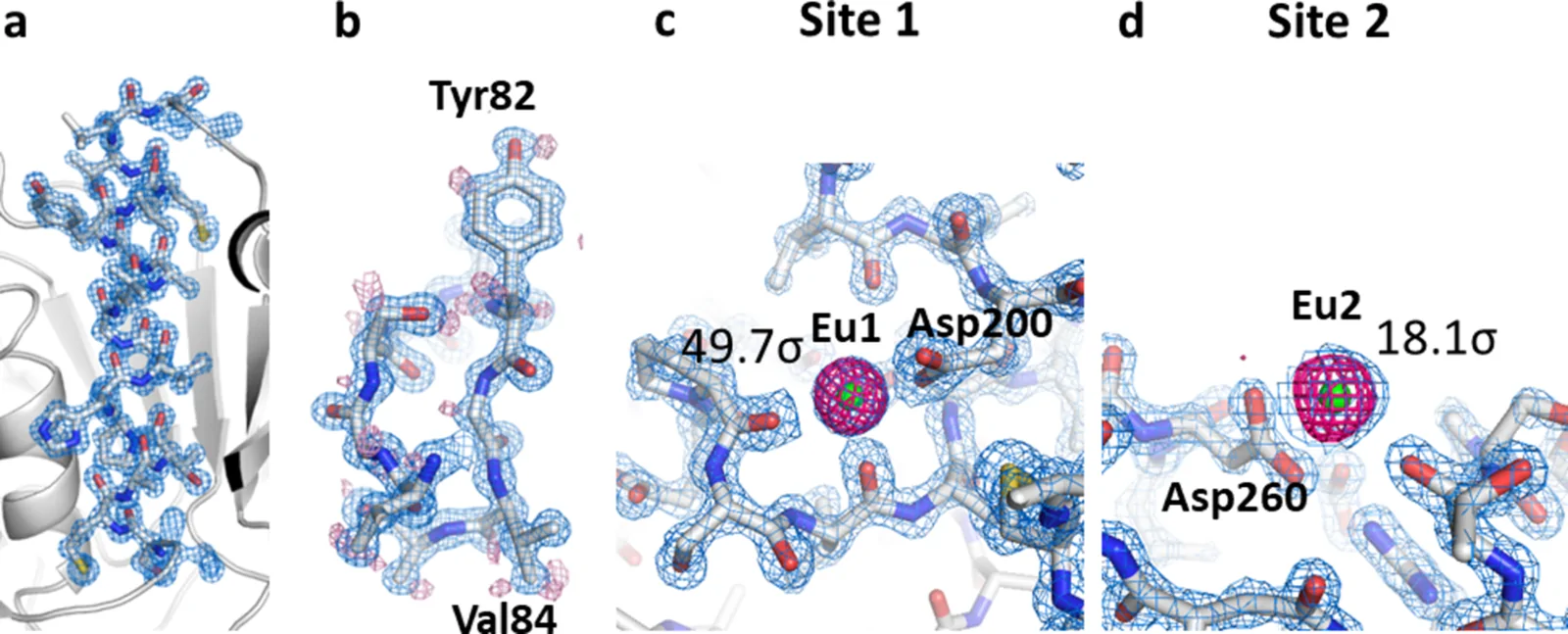
Electron density maps of proteinase K. (*a*, *b*) Close-up views of the proteinase K structure with a 2*F*_o_ − *F*_c_ electron-density map contoured at the 2.0σ level (coloured blue) and (*b*) the *F*_o_ − *F*_c_ electron-density map contoured at the 2.2σ level (coloured pink). (*c*, *d*) Close-up views of Eu ion binding sites with 2*F*_o_ − *F*_c_ electron density maps contoured at the 2.0σ level (coloured blue). Bound Eu ions are depicted as magenta spheres. The anomalous difference Fourier maps using all indexed images (contoured at the 3.5σ level) are shown in magenta.

We then investigated *de novo* phasing from the dataset of Eu-derivatized proteinase K, collected in this study. Single-wavelength anomalous diffraction (SAD) phasing was carried out using *SHELXD* and *SHELXE* (Sheldrick, 2010 ▸). The software and procedures employed for *de novo* phasing in this study were identical to those used in the Pr-derivative experiment (Sugahara *et al.*, 2020 ▸). We successfully identified two Eu ions in the asymmetric unit and subsequently solved the substructure (Fig. 4 ▸). The two Eu-binding sites corresponded to those previously identified for Pr ions in the proteinase K structure (Sugahara *et al.*, 2017 ▸; Sugahara *et al.*, 2020 ▸). We employed the coordinates of the heavy atoms for both refinement and phase calculation at a resolution of 1.7 Å in *SHEXLE*, which also automatically traced a polyalanine model of proteinase K. Subsequently, *ARP*/*wARP* (Langer *et al.*, 2008 ▸) automatically modelled 99% (276 of 279 residues) of the structure with side chains. Finally, we refined the structure at a resolution of 1.20 Å, yielding*R*/*R*_free_ values of 16.9%/18.3%. We were able to observe clear electron density maps of proteinase K [Fig. 4 ▸(*a*)]. While a higher-resolution structure is needed to accurately observe hydrogen atoms, some hydrogen atoms may be detectable in our crystal structure [Fig. 4 ▸(*b*)]. The electron density corresponding to hydrogen atoms was barely detectable at a contour level of 2.2σ.

In the final anomalous difference Fourier maps, generated from all indexed images, we observed significant anomalous peak heights of 49.7σ and 18.1σ for the two Eu atoms [Table 2 ▸, Figs. 4 ▸(*c*) and 4(*d*)]. Thus, the PEEK film and the grease matrix were found to cause minimal interference with the detection of anomalous signals in protein crystals. In the previous injection SFX study using the DATPE matrix, anomalous scattering signals were detected from the Pr atoms in the proteinase K structure (Sugahara *et al.*, 2020 ▸) from ∼23000 indexed images. The averaged anomalous densities for the two Pr ions were 46.6σ for site 1 and 27.9σ for site 2, according to *ANODE* (Table 2 ▸). Notably, there were no discernible differences in the anomalous difference Fourier maps for the heavy atoms between the two methods, while the resolution achieved in the previous data from 0.8 µm sized crystals was limited to 1.65 Å resolution (Table 2 ▸). Thus, we successfully detected anomalous signals in Eu-derivatized proteinase K crystals using the proposed fixed-target approach.

### 2.3. Simplicity and adaptability of the fixed-target procedure with grease matrix

**Figure 5:**
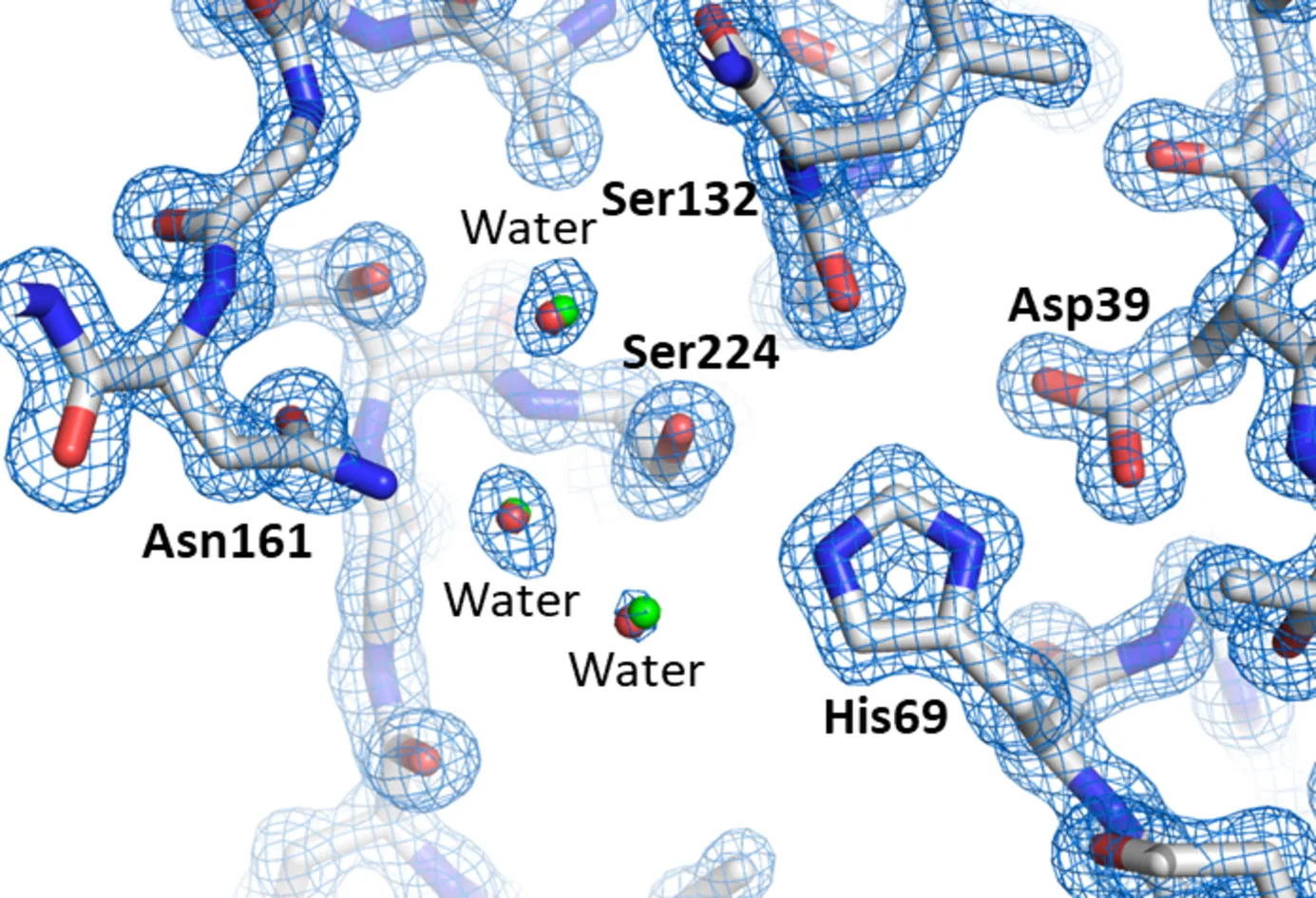
Electron density maps around catalytic site in proteinase K structure. Water molecules are depicted as red spheres for this study by the fixed-target method and green spheres for the previous study by the injection method (Masuda *et al.*, 2017 ▸). The 2*F*_o_ − *F*_c_ map contoured at the 1.5σ level is shown by blue meshes.

In the fixed-target method, the sample is spread onto a PEEK film and mounted on the sample stage for data collection. By contrast, the injection method requires loading the matrix into a cartridge integrated into the injector (Shimazu *et al.*, 2019 ▸), which generates a stable sample stream aligned with the FEL interaction point. Thus, the fixed-target approach involves a simpler sample-loading procedure with fewer steps. Nevertheless, this method raised concerns about crystal dehydration during 2D scanning for XFEL exposure. A comparison of the number of water molecules in the structures obtained using the fixed-target and injection methods showed similar counts, with 257 and 219 molecules, respectively (Table 2 ▸). The water molecules around the active site (Masuda *et al.*, 2017 ▸) were observed in a similar manner (Fig. 5 ▸). A comparison of parameters obtained from the injection and fixed-target methods showed no significant differences in unit-cell volume (Table 2 ▸). In addition, although the average *B*-factors of solvent molecules were slightly lower in the fixed-target method, they were overall comparable between the two methods. Although this comparison is limited to proteinase K, the agreement in lattice parameters and solvent *B*-factors suggests that crystal hydration is well preserved under the present experimental conditions. Physical damage to crystals may occur during mixing with the grease matrix or when spreading grease-containing crystals onto the PEEK film. Although thin spreading is important for reducing X-ray background scattering, it may introduce mechanical stress depending on the crystal properties. While grease matrices have been successfully used for various crystals (Sugahara *et al.*, 2015 ▸; Nakane *et al.*, 2015 ▸; Yamashita *et al.*, 2015 ▸; Colletier *et al.*, 2016 ▸; Sugahara *et al.*, 2016 ▸; Fukuda *et al.*, 2016 ▸; Nakane, Hanashima *et al.*, 2016 ▸; Edlund *et al.*, 2016 ▸; Suga *et al.*, 2017 ▸; Sugahara *et al.*, 2017 ▸; Yamashita *et al.*, 2017 ▸; Naitow *et al.*, 2017 ▸; Hutchison *et al.*, 2017 ▸; Woitowich *et al.*, 2018 ▸; Maestre-Reyna *et al.*, 2022 ▸; Maestre-Reyna *et al.*, 2023 ▸; Li *et al.*, 2024 ▸), other approaches (Zarrine-Afsar *et al.*, 2012 ▸; Hunter *et al.*, 2014 ▸; Mueller *et al.*, 2015 ▸; Murray *et al.*, 2015 ▸; Oghbaey *et al.*, 2016 ▸; Baxter *et al.*, 2016 ▸; Doak *et al.*, 2018 ▸; Tolstikova *et al.*, 2019 ▸; Monteiro *et al.*, 2019 ▸; Lee, Lee *et al.*, 2020 ▸; Nam *et al.*, 2021 ▸) may be preferable for samples that are fragile under mechanical stress.

Furthermore, the fixed-target procedure does not need crystal-size filtering. In the injection method, matrix viscosity is critical in maintaining a continuous and stable sample stream from the nozzle of a high-viscosity micro-extrusion injector during serial sample loading. A viscosity-adjustable matrix is necessary to maintain a stable sample stream for the injection method (Sugahara *et al.*, 2020 ▸). In contrast, the fixed-target method does not demand precise adjustments to matrix viscosity. However, a low-viscosity matrix may cause crystal sinking on the PEEK film (Lee, Lee *et al.*, 2020 ▸). In this case, we employed a 15% (*w*/*w*) DATPE grease matrix to keep the crystals in fixed positions during data collection.

Our fixed-target sample holder is compatible with the capacity for sample rotation, based on a previously developed model (Takaba *et al.*, 2023 ▸; Takaba *et al.*, 2024 ▸). In this study, however, we collected a data set without rotation. Proteinase K crystals sized 10 µm were randomly distributed within a matrix of ∼200 ± 100 µm thickness. The thickness of the sample layer can increase background scattering and affect the uniformity of pump-laser illumination (Niziński *et al.*, 2025 ▸). To achieve a more consistent layer and minimize both background variability and fluctuations in excitation levels, it is important to improve the method for spreading the sample onto the substrate. Such improvements could include using a wide spatula during application to the film or slightly reducing the viscosity of the grease matrix while maintaining the position of the crystals. Crystals smaller than 30 µm are expected to exhibit random orientation within a matrix of ∼100 µm thickness (Sugahara *et al.*, 2015 ▸). The assumption that crystals smaller than 30 µm exhibit random orientation was supported by a comparison of orientation matrices between the fixed-target and extrusion datasets. Compared with the injection method, the grease matrix in the fixed-target method tends to have greater variability in thickness, and regions with higher multi-hit rates may occur. However, with the sample volume used in this study (50 µL), no significant statistical differences were observed (*cf*. Eu- and Pr-derivatives in Table 2 ▸). Therefore, we consider approximately 50 µL to be an appropriate amount for applying the grease matrix onto the film. When using larger volumes, there is a higher likelihood of multi-hit events, and caution should be exercised. For crystals with preferential orientations, the sample stage is initially aligned to the eucentric position and subsequently tilted from 60° to 0° around the φ axis during XFEL exposure (Fig. 1 ▸) (Takaba *et al.*, 2024 ▸). Thus, this approach offers high adaptability and has the potential to improve the acquisition of high-quality data from various challenging targets.

Silicon-based fixed-target chips have the significant advantage of high precision and reproducibility in microfabrication, enabling dense crystal packing with well structures or through-hole arrays, and precise positioning (Zarrine-Afsar *et al.*, 2012 ▸; Hunter *et al.*, 2014 ▸; Mueller *et al.*, 2015 ▸; Murray *et al.*, 2015 ▸; Oghbaey *et al.*, 2016 ▸; Tolstikova *et al.*, 2019 ▸). This feature is especially effective for micro-focused X-ray beams, allowing high-precision irradiation control per crystal and time-resolved measurements with triggering. Furthermore, silicon exhibits high thermal conductivity and dimensional stability, making it suitable for experiments where temperature control is critical (Hsiao *et al.*, 2006 ▸). On the other hand, polymer-based substrates such as the PEEK film used in our method offer advantages including flexibility, ease of fabrication, cost efficiency, and low background scattering of X-rays (Thorne *et al.*, 2003 ▸), while exhibiting high resistance to hard X-ray irradiation, in contrast to brittle materials like silicon nitride. Additionally, the encapsulation of crystals within a grease matrix provides versatile compatibility with various sample types without requiring specialized microfabrication. Nevertheless, when performing time-resolved experiments with high-viscosity media, the transmittance needs to be carefully evaluated (Niziński *et al.*, 2025 ▸; Malla *et al.*, 2025 ▸). Furthermore, the general applicability of this method should be assessed using more fragile or lower-symmetry samples. Fixed-target pump–probe SFX can suffer from optical light contamination (Gotthard *et al.*, 2024 ▸). Nevertheless, a key advantage of fixed-target methods is their low sample consumption (Manna *et al.*, 2025 ▸). Thus, the choice of fixed-target support depends on the measurement objectives and experimental conditions. Silicon substrates are advantageous when prioritizing high-density crystal packing and precise single-crystal irradiation, whereas polymer film-based methods are more suitable when emphasizing ease of device fabrication and operation, simpler sample preparation, and reduced background scattering.

## 3. Conclusion

This study has introduced a fixed-target protein SFX method, utilizing the grease matrix and the large-area support film. This approach significantly reduces the time and labour required for sample loading and preparation while minimizing sample consumption and dependence on crystal size. It has also been demonstrated that neither the matrix nor the sample support film interfere with the detection of anomalous signals and hydrogen atoms in protein crystals, enabling high-quality structure analysis, without detectable dehydration during data collection. This adaptation enhances the versatility of the technique, making it applicable to a broad range of samples, including not only small organic molecular crystals but also protein crystals. The femtosecond pump laser used at SACLA has a spot size of approximately 42 µm (FWHM) on the sample surface (Togashi *et al.*, 2020 ▸). This is larger than the XFEL pulse spacing of about 25 µm used in our fixed-target experiments, which may lead to optical overlap and cross-excitation between adjacent crystals. To mitigate this issue, the irradiation spacing should be set to approximately twice the spot size, that is, about 50 µm. Although this adjustment theoretically reduces data collection efficiency, it still allows for sufficient data acquisition. In the future, advances in laser focusing technology are expected to reduce the spot size, allowing narrower spacing and thereby decreasing sample consumption.

## 4. Materials and methods

### 4.1. Sample preparation

We prepared proteinase K (No. P2308, Sigma) crystals sized 10 µm × 10 µm following the previously reported protocols (Sugahara *et al.*, 2016 ▸; Yazawa *et al.*, 2016 ▸). After centrifuging a 100 µl crystal sample of the storage solution (with a crystal density of 4.8 × 10^7^ crystals ml^−1^) at ∼1300*g*–3000*g* for 10 s using a compact tabletop centrifuge, we removed a 90 µl aliquot of the supernatant. Subsequently, we added a 10 µl sample of the concentrated crystal solution to a 90 µl heavy-atom solution, comprising 27.8 m*M* EuCl_3_ (HR2-450-8, Hampton Research), 0.5 *M* NaNO_3_, and 0.1 *M* MES–NaOH (pH 6.5). We then incubated the crystal solution at 20°C for 90 min. After centrifuging a 100 µl crystal sample of the heavy-atom solution for 10 s, we removed a 90 µl aliquot of supernatant [Fig. 2 ▸(*a*)]. For the grease matrix, we employed a 15% (*w*/*w*) dextrin palmitate/di­alkyl tetra­phenyl ether oil (DATPE) grease (Sugahara *et al.*, 2020 ▸). We dispensed a 10 µl aliquot of the crystal suspension into 90 µl of 15% (*w*/*w*) dextrin palmitate/DATPE grease on a glass slide, then mixed them with a spatula [Figs. 2 ▸(*b*) and 2(*c*)]. The PEEK film (Urban Crown Co Ltd, 2000-012G, thickness 12 µm) (Hasegawa *et al.*, 2021 ▸) was fixed to the sample holder window using cellophane tape [Fig. 2 ▸(*d*)]. This simple approach ensured adequate adhesion during the experiments and allowed easy replacement of the film. A ∼50 µl aliquot of the matrix was evenly spread over the film using a spatula [Fig. 2 ▸(*e*)].

### 4.2. Data collection

We carried out data collection at SACLA (Ishikawa *et al.*, 2012 ▸; Yabashi *et al.*, 2015 ▸; Tono *et al.*, 2019 ▸) BL2 in experimental hutch 3 using femtosecond X-ray pulses with a wavelength of 0.827 Å (15 keV), and a pulse energy of approximately 200 µJ. Each X-ray pulse delivered ∼10^12^ photons within a 10 fs duration (FWHM) to the sample. The X-ray beam was focused via Kirkpatrick–Baez mirrors (Yumoto *et al.*, 2013 ▸) to achieve a spot size of 1.5 µm × 1.5 µm. The crystals were kept at approximately 25°C in the experimental hutch. Diffraction images were recorded at 30 Hz through 2D scanning of the matrix sample using a Rayonix MX300-HS detector operating in 4-by-4 binning mode (Fig. 1 ▸). The sample stage allowed for movement along the *XYZ* axes and rotation around the φ axis (Takaba *et al.*, 2023 ▸; Takaba *et al.*, 2024 ▸). The sample stage motors used in the experiment were pulse-type motor drivers (Melec, model H750v1/GDB-5F40). The sample plate was scanned with XFEL pulses by moving the stage in the *XZ* plane at a speed of 750 µm s^−1^ (25 µm × 30 Hz).

### 4.3. Structure determination

Bragg-spot-containing image files were identified using the diffraction data processing program *DIALS* version 3.5.0 (Winter *et al.*, 2018 ▸). Frames exhibiting more than ten identified spots were converted to the HDF format using *Python* with the *h5py* package, and subsequently processed with the *CrystFEL* suite version 0.10.2 for indexing and integration of the intensities (White *et al.*, 2012 ▸). We followed procedures adapted for the SACLA data acquisition system (Joti *et al.*, 2015 ▸; Nakane, Joti *et al.*, 2016 ▸). We determined diffraction peak positions using the *peakfinder8* algorithm (Barty *et al.*, 2014 ▸) and passed them to *MOSFLM* (Powell, 1999 ▸) or *DirAx* (Duisenberg, 1992 ▸) for indexing. We applied no sigma cutoff or saturation cutoff. We merged the measured diffraction intensities using *process_hkl* in the *CrystFEL* suite (version 0.10.2) with the scaling (--*scale*) option available in this version (White *et al.*, 2012 ▸). For the Eu-derivatized proteinase K, we carried out substructure search, phasing, and phase improvement using the *SHELX C*, *D* and *E* programmes (Sheldrick, 2010 ▸). We fed the auto-traced model from *SHELXE* into *ARP*/*wARP* (Langer *et al.*, 2008 ▸) in the *CCP4* suite (Collaborative Computational Project, Number 4, 1994 ▸). We performed manual model revision and structure refinement using *Coot* (Emsley & Cowtan, 2004 ▸) and *PHENIX* (Adams *et al.*, 2010 ▸), respectively. In Table 1 ▸, we summarize details of the data collection and refinement statistics. The anomalous peak heights of the two Eu atoms were calculated using *ANODE* (Thorn & Sheldrick, 2011 ▸) and are shown in Fig. 4 ▸. Figs. 4 ▸ and 5 ▸ were prepared using *PyMOL* (https://www.pymol.org).

## Figures and Tables

**Table 1:** Crystallographic statistics

Data collection	
Space group	*P*4_3_2_1_2
Unit-cell parameter	
*a* = *b* (Å)	68.70
*c* (Å)	108.98
Number of collected images	114977
Number of hits	30692 (27%)
Number of indexed images	20763 (68%)
Number of total reflections	21761450
Number of unique reflections	82104
Resolution range (Å)	29.6–1.2 (1.22–1.20)†
Completeness (%)	100 (100)‡
Multiplicity	265 (180)‡
*R*_split_ (%)§	13.0 (82.0)‡
CC_1/2_	0.940 (0.472)‡
〈*I*/σ(*I*)〉	6.0 (1.4)‡

Refinement	
*R*/*R*_free_ (%)	16.9/18.3
R.m.s.d., bond lengths (Å)	0.0054
R.m.s.d., angles (°)	0.83
Average *B* factor (Å^2^)	
Protein	16.17
Water	28.48
Ramachandran statistics (%)	
Favoured	91.1
Allowed	8.9
Outliers	0
PDB code	9kw1

† Values in parentheses indicate the resolution range of the outermost shell.

‡ Values in parentheses are for the outermost shell.

§ *R*_split_ = 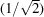

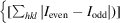
 / 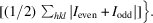

**Table 2:** Comparison of fixed-target and injection methods in diffraction experiments of proteinase K

	Fixed target	Injection	Injection
Number of collected images	114977	137167	362074
Number of hits	30692 (27%)	36818 (27%)	158777 (44%)
Number of indexed images	20763 (68%)	23406 (64%)	82074† (52%)
Unit-cell volume (Å^3^)	5.1 × 10^5^	5.0 × 10^5^	5.1 × 10^5^
Resolution range (Å)	29.6–1.20 (1.22–1.20)‡	32.5–1.65 (1.68–1.65)‡	27.2–1.20 (1.23–1.20)‡
CC_1/2_ in outermost shell	0.47	0.42	0.47
〈*I*/σ(*I*)〉 in outermost shell	1.4	1.1	1.3
Number of water molecules in asymmetric unit	257	180	219
Average *B*-factor for water (Å^2^)	28.48	33.69	37.97
Heavy atoms	Eu	Pr	–

Anomalous peak heights obtained from *ANODE*			
Site 1	49.7	46.6	–
Site 2	18.1	27.9	–
Total amounts of protein used (mg)	∼0.2	∼0.2	∼1.4–1.9
PDB code	9kw1	6k2x	5kxu
Reference	This work	Sugahara *et al.* (2020 ▸)	Masuda *et al.* (2017 ▸)

† Number of used images.

‡ Values in parentheses indicate the resolution range of the outermost shell.
